# DeepRefiner: high-accuracy protein structure refinement by deep network calibration

**DOI:** 10.1093/nar/gkab361

**Published:** 2021-05-17

**Authors:** Md Hossain Shuvo, Muhammad Gulfam, Debswapna Bhattacharya

**Affiliations:** Department of Computer Science and Software Engineering, Auburn University, Auburn, AL 36849, USA; Department of Computer Science and Software Engineering, Auburn University, Auburn, AL 36849, USA; Department of Computer Science and Software Engineering, Auburn University, Auburn, AL 36849, USA; Department of Biological Sciences, Auburn University, Auburn, AL 36849, USA

## Abstract

The DeepRefiner webserver, freely available at http://watson.cse.eng.auburn.edu/DeepRefiner/, is an interactive and fully configurable online system for high-accuracy protein structure refinement. Fuelled by deep learning, DeepRefiner offers the ability to leverage cutting-edge deep neural network architectures which can be calibrated for on-demand selection of adventurous or conservative refinement modes targeted at degree or consistency of refinement. The method has been extensively tested in the Critical Assessment of Techniques for Protein Structure Prediction (CASP) experiments under the group name ‘Bhattacharya-Server’ and was officially ranked as the No. 2 refinement server in CASP13 (second only to ‘Seok-server’ and outperforming all other refinement servers) and No. 2 refinement server in CASP14 (second only to ‘FEIG-S’ and outperforming all other refinement servers including ‘Seok-server’). The DeepRefiner web interface offers a number of convenient features, including (i) fully customizable refinement job submission and validation; (ii) automated job status update, tracking, and notifications; (ii) interactive and interpretable web-based results retrieval with quantitative and visual analysis and (iv) extensive help information on job submission and results interpretation via web-based tutorial and help tooltips.

## Graphical Abstract

**Graphical Abstract:**
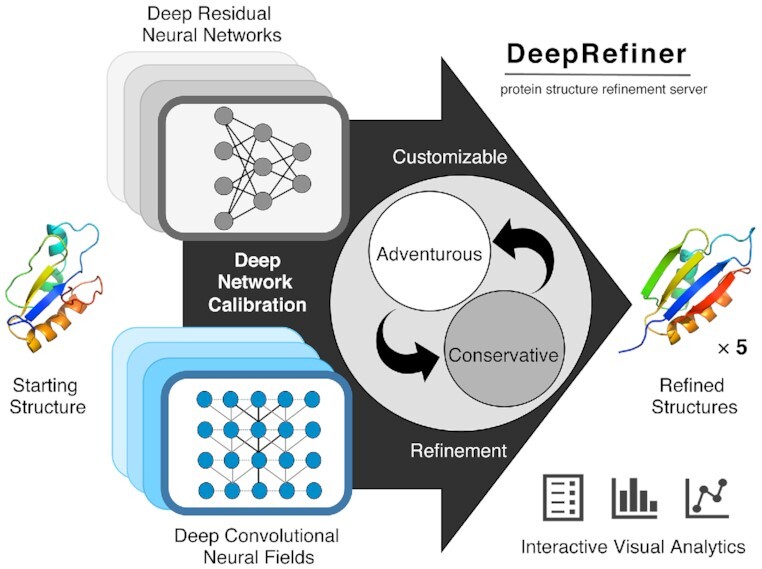
DeepRefiner webserver for high-accuracy protein structure refinement by deep network calibration.

## INTRODUCTION

Deep learning has transformed protein structure prediction. Recent editions of the Critical Assessment of Techniques for Protein Structure Prediction (CASP) experiments have witnessed a major breakthrough in accurately predicting the structure of a protein from sequence information through the application of advanced deep neural network architectures (1–3). However, a predicted structure can still deviate from the experimental structure in terms of the accuracy of the backbone positioning or the side-chain conformation or both (4). The goal of protein structure refinement is to increase the accuracy of such a moderately accurate starting structure by driving it towards the experimental quality. Some of the most successful approaches for structure refinement rely on large-scale conformational search for low energy structures (5–7), which are time-consuming and computationally expensive. To make structure refinement both accurate and fast, we proposed refineD (8), which employed deep neural networks to estimate residue-level errors from a starting structure and then minimized the cumulative error through inexpensive energy-minimization-based restrained relaxation for improved structure refinement. Due to the advantages associated with computationally efficient energy minimization guided by deep learning over time-consuming conformational search, several recent studies have sought to guide refinement using deep learning (9,10); even though the full-fledged versions of these methods are not yet publicly available. As such, a robust and publicly accessible webserver that can perform high-accuracy structure refinement in a computationally efficient manner guided by deep learning has the potential for broad dissemination and a field-wide impact. With rapid new developments in the field, however, the residue-level error estimators used in our original refineD method no longer represents the state of the art. In particular, the recent CASP experiments (4) have witnessed significant new progress in inter-residue distance prediction through cutting-edge deep neural network training in conjunction with metagenomic sequencing. Thus, integrating distance information for improved residue-level error estimation combined with the power of state-of-the-art deep learning models is critically important to further improve protein structure refinement. Moreover, the ability to leverage deep network architectures that can be calibrated for on-demand selection of adventurous or conservative refinement modes targeted at degree or consistency of refinement, can enhance the versatility of such a method to a wide range of use cases.

Here, we present DeepRefiner, an interactive and fully configurable webserver for high-accuracy protein structure refinement by deep network calibration. DeepRefiner first estimates residue-level errors from a starting structure using an ensemble of advanced deep neural network architectures and subsequently minimizes the cumulative error through energy-minimization-based restrained relaxation, leading to five refined structures. The advanced error estimation module in DeepRefiner employs a high-resolution version of our successful application of very deep and fully convolutional residual neural networks (11) for distance-based protein model quality estimation (12) at finer-grained error thresholds trained specifically for structure refinement. DeepRefiner offers an interactive user interface that takes a starting structure in PDB format as input and outputs five refined structures along with their global and local quality estimations, comparison to the starting structure, and breakdown of residue-wise structural features. The customizable DeepRefiner interface provides (i) choice of cutting-edge deep neural network architectures for estimating residue-level errors including deep conditional neural fields and deep residual neural networks; (ii) on-demand selection of adventurous or conservative refinement mode by calibrating the ensemble of deep networks; (iii) comprehensive post-refinement analysis using MolProbity (13), GOAP (14), OPUS-PSP (15), DFIRE (16) and RWplus (17); (iv) fully automated job status update, tracking and notifications; (v) interactive and interpretable web-based results and (vi) extensive help information on job submission and results interpretation via web-based tutorial and help tooltips. Our method was rigorously tested in the most recent CASP refinement experiments (18) under the group name ‘Bhattacharya-Server’ and was officially ranked as the No. 2 refinement server in CASP13 (second only to ‘Seok-server’ and outperforming all other refinement servers) and No. 2 refinement server in CASP14 (second only to ‘FEIG-S’ and outperforming all other refinement servers including ‘Seok-server’). DeepRefiner webserver is freely available at http://watson.cse.eng.auburn.edu/DeepRefiner/.

## MATERIALS AND METHOD

### Overview of the DeepRefiner pipeline

**Figure 1.**
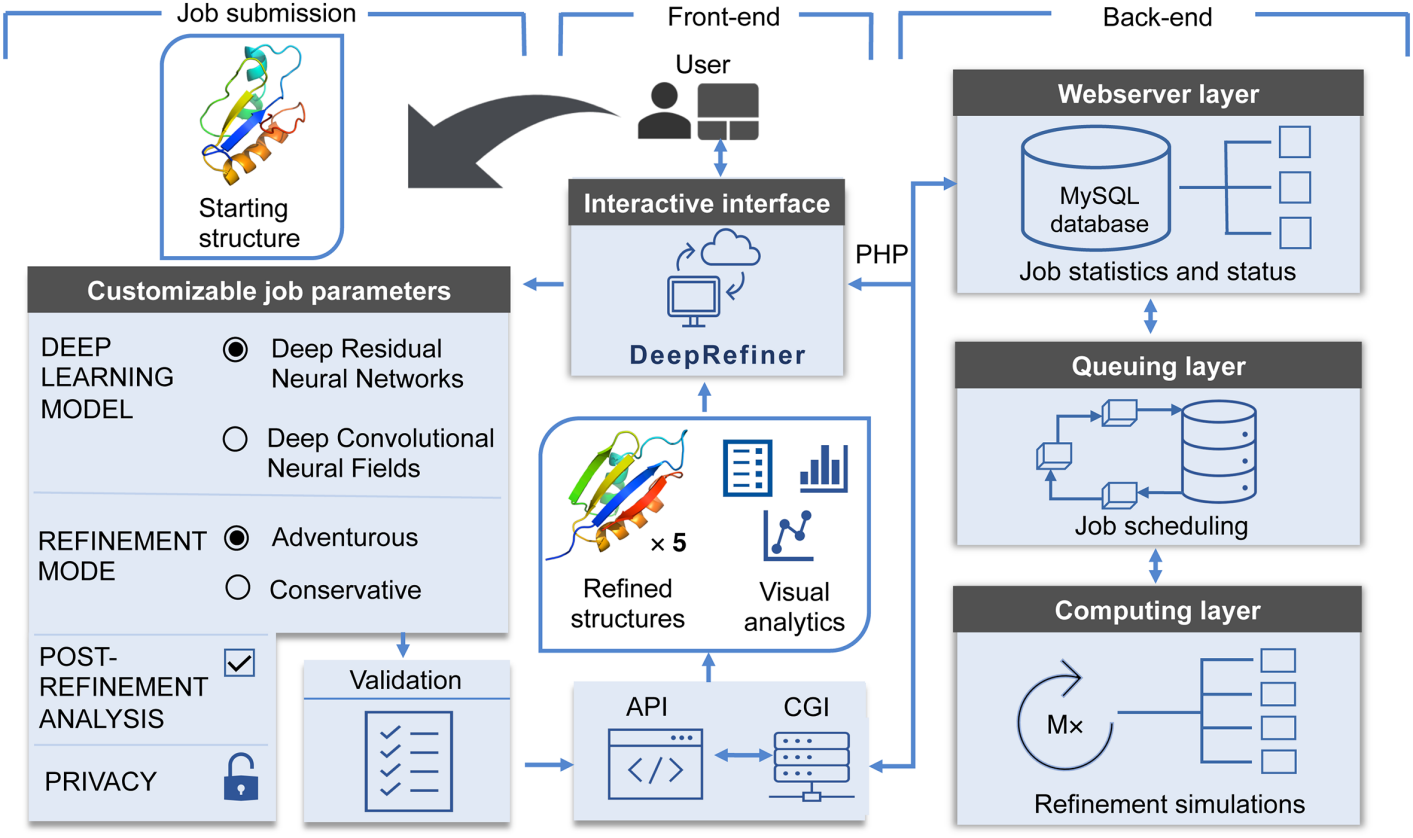
The flowchart of the DeepRefiner pipeline consisting of the webserver front-end module for submitting customizable refinement jobs and retrieving the results through the interactive web interface, and the back-end module that processes the refinement jobs.

Figure 1 shows the flowchart of the DeepRefiner pipeline consisting of the webserver front- and back-end modules. The front-end module offers an interactive web-based interface that lets the user submit customizable refinement jobs, readily processes and validates user inputs, dynamically shows the job status and progress, presents statistics of the processed job, and provides interactive quantitative and visual analysis of the results; while the back-end module executes the refinement jobs. Users can choose to protect the privacy of their jobs by submitting a private refinement job where the refinement results will only be accessible to the submitter.

#### Front-end module

The front-end module of the webserver provides a web-based job submission interface where the user needs to provide only a starting structure in PDB format and a job name as mandatory inputs. The interface offers a wide range of options for customizing a refinement job including the ability to select deep network architectures, calibrate the refinement modes for on-demand selection of adventurous or conservative refinement targeted at degree or consistency of refinement, perform comprehensive post-refinement analysis, and protect job privacy. Two deep neural network architectures for residue-level error estimation are available, both independently supporting on-demand selection of adventurous or conservative refinement modes by calibrating the model ensemble. The submitted refinement job is then dynamically validated for consistency and passed on to the back-end via Common Gateway Interface (CGI).

#### Back-end module

The back-end of the webserver consists of three sequentially interdependent layers including the webserver layer, queuing layer, and computing layer. The webserver layer directly interacts with the front-end and maintains job statistics and status using a MySQL database. The queuing layer performs job scheduling by implementing a first-in-first-out (FIFO) job queue. Additionally, it continuously interacts with the webserver layer to dynamically update job status and subsequently communicates with the front-end through CGI. Once the queuing layer releases a job for execution, the job starts running in the computing layer. The computing layer executes the job based on the supplied job parameters by first employing the selected deep neural network architecture for estimating the residue-level errors from the starting structure and then performing either adventurous or conservative refinement through energy-minimization-based restrained relaxation by calibrating the chosen model ensemble to generate five refined structures. After completion of the refinement job, the webserver performs comprehensive post-refinement analysis on the refined structures by estimating their global and local qualities; evaluating and reporting the scores of several knowledge-based statistical potentials including GOAP, OPUS-PSP, DFIRE, and RWPlus (14–17); performing MolProbity (13) analysis for assessing the physical realism; and comparing to the staring structure in terms of backbone and side-chain positioning as well as the consistencies between structural properties such as secondary structure and solvent accessibility. The results are returned to the front-end module for interactive and interpretable web-based quantitative and visual analytics (see Supplementary Figure S1) with an email notification sent to the user, if an email address is provided.

### Deep network calibration

#### Architectures of the deep learning models

DeepRefiner offers the choice of two deep neural network architectures for estimating residue-level errors including deep conditional neural fields (DeepCNF) (19,20) and deep residual neural networks (ResNet) (11). DeepCNF architecture was employed in our original refineD method (8) to classify every residue of the starting structure to be within four fine-grained error thresholds of 0.5, 1, 2 and 4 Å by independently training an ensemble of four DeepCNF classifiers. Collectively, the set of four classifiers results in residue-level ensemble error classifications. The featurization for representing the residues in the starting structure includes sequence profile, consistency between predicted and observed structural properties (secondary structure and solvent accessibility), and biophysical energy terms. Our newly trained ResNet ensemble classifiers incorporate distance information as additional features to perform residue-level ensemble error classifications at the same fine-grained error thresholds of 0.5, 1, 2 and 4Å (see the detailed description in Text S1 in the Supporting Information, SI**)**. The ResNet classifiers represent a high-resolution version of our successful application of distance-based protein model quality estimation (12), while specifically targeting finer-grained error thresholds for structure refinement (see Text S2).

#### Calibrating the model ensemble

The residue level ensemble error estimates are then converted into multi-resolution probabilistic restraints weighted by their associated likelihood values and applied on the C_α_ atom of the starting structure in the form of Rosetta Coordinate Constraint with FLAT_HARMONIC function at 0.5, 1, 2 and 4Å thresholds in conjunction with the REF15 scoring function of Rosetta (21). Subsequently, energy-minimization-based restrained relaxation is iteratively employed for structure refinement (see Text S3). All restraints corresponding to the four thresholds can be simultaneously applied in a cumulative manner for conservative refinement mode aimed at achieving consistently positive refinement. Alternatively, restraints can be applied in a non-cumulative manner independent of each other for adventurous refinement mode aimed at producing higher degree of structural changes. The global and local qualities of the resulting refined models can be estimated via probabilistic combination of the ensemble classifiers (see Text S4). In summary, calibration of the model ensemble controls the characteristics of the restrained relaxation, thus affecting the degree of conformational change that can be used for achieving on-demand conservative or adventurous structure refinement.

## RESULTS

### Blind performance assessment in CASP

The refinement protocol employed in DeepRefiner has been extensively tested in the refinement category of CASP13 and CASP14 in a strictly blind manner under the group name ‘Bhattacharya-Server’ and was ranked highly among all refinement servers. Table 1 shows the performance comparison of ‘Bhattacharya-Server’ with other participating server groups based on top-ranked submission under the refinement category of CASP13 and CASP14 in terms of the sum of overall Z-scores calculated as the weighted sum of Z-scores for GDT-HA (22), GDC-sc (23), RMSD (24), SphereGrinder (25) and MolProbity (13), following the same methodology adopted in prior CASP refinement assessment (26) (see **Text S5**). ‘Bhattacharya-Server’ was officially ranked No. 2 among all other refinement server groups in both CASP13 (second only to ‘Seok-server’ and outperforming all other refinement servers) and CASP14 (second only to ‘FEIG-S’ and outperforming all other refinement servers including ‘Seok-server’). We report the per-target *Z*-score in the supplementary information (see Supplementary Tables S1 and S2 for per-target Z-scores broken down by each accuracy metric involved in the calculation of the overall *Z*-score). We further analyze the degree of structural refinement attained by ‘Bhattacharya-Server’ for CASP13 and CASP14 refinement targets in terms of various accuracy measures including GDT-HA, GDC-sc, and MolProbity scores with respect to length and accuracy of the starting structures (in terms of GDT-HA) considering the best submission. The results demonstrate that most promising refinement cases are generally observed for smaller targets having length less than 100 residues and those in the medium range of starting accuracies having starting GDT-HA scores between 40 and 60 units (see Supplementary Figures S2 and S3). The DeepCNF-based error estimation module used in ‘Bhattacharya-Server’ for both CASP13 and CASP14 shall further improve due to the incorporation of advanced ResNet-based ensemble error classifiers, ultimately improving the refinement performance of DeepRefiner.

**Table 1.** Performance comparisons of server groups participating in the refinement category of CASP13 and CASP14. Groups are sorted by descending sum of overall *Z*-scores

	Group name	Group #	Sum overall Z-score	Rank sum overall *Z*-score
CASP13	Seok-server	156	21.686	1
	Bhattacharya-Server	102	13.125	2
	YASARA	004	12.976	3
	MUFold_server	312	10.895	4
	3DCNN	359	0.701	5
CASP14	FEIG-S	013	35.344	1
	Bhattacharya-Server	149	21.822	2
	Seok-server	070	18.404	3
	MULTICOM-CLUSTER	075	12.312	4
	MUFOLD	081	4.178	5

### Case study

**Figure 2.**
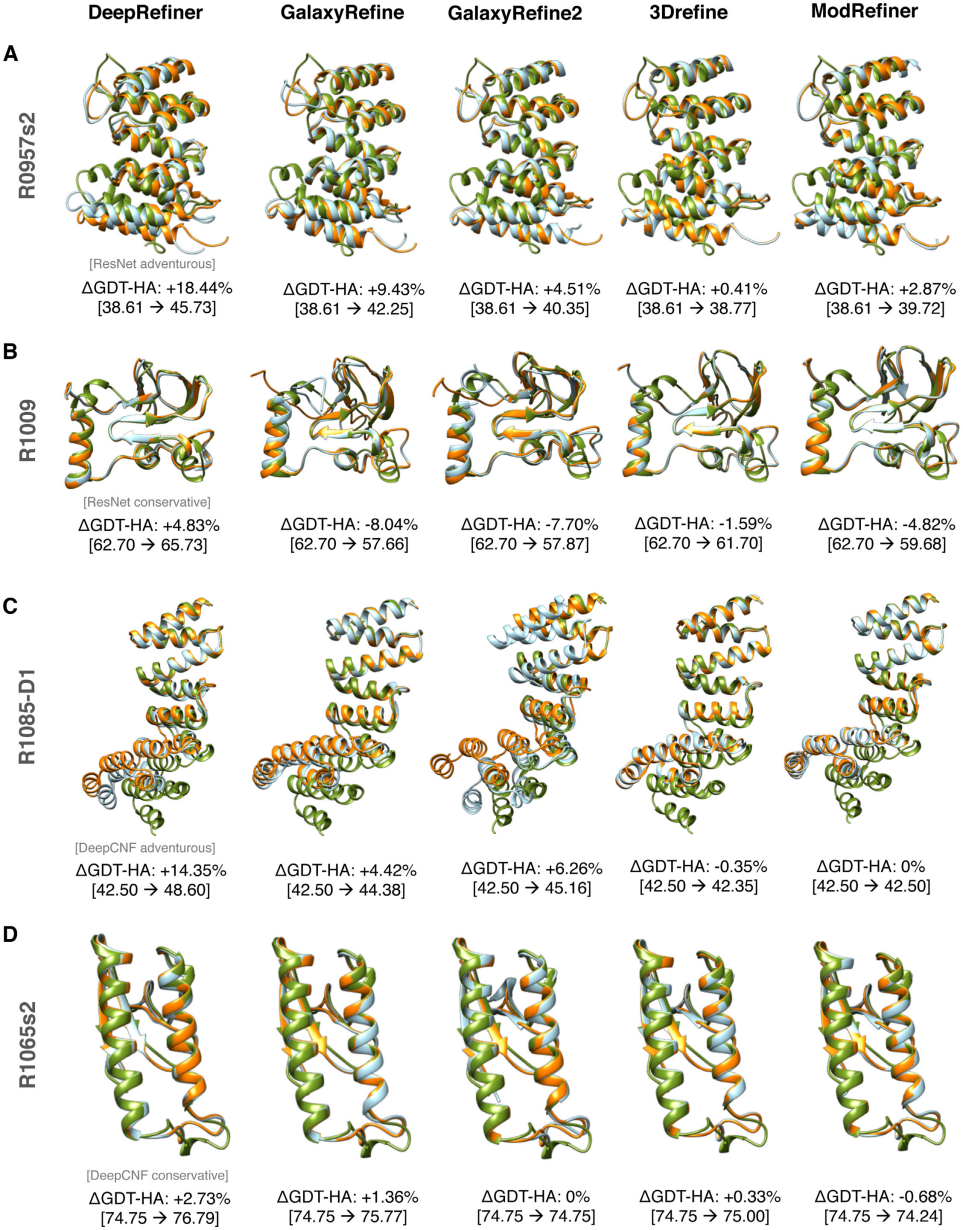
Representative refinement examples from four CASP refinement targets. DeepRefiner yields better refinement than other methods by deep network calibration using either ResNet- (**A**) R0975s2 and (**B**) R1009; or DeepCNF-based error estimation (**C**) R1085-D1 and (**D**) R1065s2.

In Figure 2, we present the refinement results for four representative CASP targets including two targets R0957s2 and R1009 from CASP13; and two targets R1085-D1 and R1065s2 from CASP14. DeepRefiner alternates between adventurous or conservative refinement modes by deep network calibration using either ResNet- or DeepCNF-based error estimation. We also submit these four targets to several popular refinement webservers including GalaxyRefine (27), GalaxyRefine2 (28), 3Drefine (29), and ModRefiner (30) for performance comparison. In all cases, DeepRefiner outperforms the other servers by consistently producing positive and better refinement. DeepRefiner's adventurous refinement modes lead to noticeable structural improvements with much higher degree of refinement compared to the other methods, whereas the conservative modes yield modest but positive refinement with higher consistency even when all other methods produce negative refinement.

## WEB SERVER

### Hardware and software

The server runs on a Linux cluster of 2.20-GHz Intel Xeon E5-2698 v4 20-core processors. The web application uses the PHP scripting language, JavaScript programming language and MySQL database. WebGL-based molecular visualization package 3Dmol.js (31) is used to visualize the protein structures. The DeepRefiner pipeline is implemented using Python. The webserver is compatible with most modern web browsers including Mozilla Firefox, Google Chrome, Safari, and Microsoft Edge.

### Input and output

The mandatory inputs are a job name and a starting structure for refinement in PDB format. The customizable DeepRefiner interface provides users the ability to fully configure various optional job parameters including deep learning model, refinement mode, post-refinement analysis, and job privacy. An optional email address can also be provided for automated status update of the refinement job via email. The number of residues in the starting structure is limited to 500 for computational efficiency. The average run time is several hours after the job enters the running state. Five refined structures ranked based on the estimated global qualities, MolProbity scores, and various statistical potentials are visualized in the interactive web interface and are downloadable in the PDB format. Information on structural comparison between the starting structure and the refined structures is provided in terms of GDC-sc, GDT-HA, GDT-TS and C_α_-RMSD. Structural agreement between the starting structure and the refined structures in terms of secondary structure and solvent accessibility is shown in a visually interpretable manner. Estimated local quality containing the residue-level error estimates are visualized in a graphical format for the identification of potentially unreliable local regions. The full set of results, including the refined structure and text files containing the refinement analysis, can be downloaded as a compressed zipped archive.

## CONCLUSION

DeepRefiner presents a publicly available webserver for accurate and efficient protein structure refinement. DeepRefiner leverages cutting-edge deep neural network architectures that can be calibrated for on-demand selection of adventurous or conservative refinement modes targeted at degree or consistency of refinement. The method was successful in blind refinement experiments in CASP13 and CASP14. DeepRefiner offers an interactive and versatile web interface for the submission, monitoring, results retrieval, and analysis of refinement jobs in order to drive a moderately accurate starting structure towards the experimental quality. We may further improve the accuracy of our method in particular and structure refinement in general by exploring newer deep learning models to guide refinement that can be autonomously calibrated based on the quality of the starting structure and by directly outputting Cartesian coordinates of the refined models without the need for energy-minimization-based restrained relaxation. More generally, integrated sampling and scoring from a unified deep architecture shall further improve protein structure refinement.

## DATA AVAILABILITY

DeepRefiner webserver is freely available at http://watson.cse.eng.auburn.edu/DeepRefiner/.

## Supplementary Material

gkab361_Supplemental_File
