# Supplementary material for: DeepRefiner: high-accuracy protein structure refinement by deep network calibration
Source: Nucleic Acids Res. 2021 May 17;49(W1):W147–52. doi: 10.1093/nar/gkab361 (PMC8262753; doi:10.1093/nar/gkab361)
Supplement: gkab361_Supplemental_File [file gkab361_supplemental_file.pdf]

Supplementary Information

for

DeepRefiner: high-accuracy protein structure refinement by deep network calibration

Md Hossain Shuvo[1], Muhammad Gulfam[1], Debswapna Bhattacharya[1,2,*]

[1]Department of Computer Science and Software Engineering and [2]Department of Biological Sciences, Auburn University, Auburn, AL 36849, United States of America.

*To whom correspondence should be addressed. Phone: +1 (334) 844-6321; Fax: +1 (334) 844-6329; Email: bhattacharyad@auburn.edu

Table of Contents

Supporting Text

Text S1: Feature generation for the advanced error estimation module of DeepRefiner.

Text S2: Training an ensemble of deep residual neural networks at fine-grained error thresholds.

Text S3: Iterative restrained relaxation for guiding protein structure refinement.

Text S4: Global and local quality estimation of the refined structures.

Text S5: Refinement evaluation.

Supporting Tables

Table S1. Per-target Z-scores for Seok-server (156), Bhattacharya-Server (102), YASARA (004), MUFold_server (312), and 3DCNN (359) in CASP13 in terms of GDT-HA, RMSD, GDC-sc, SphereGrinder, and MolProbity scores as well as per-target overall Z-score calculated as the weighted sum of Z-scores for GDT-HA, GDC-sc, RMSD, SphereGrinder, and MolProbity.

Table S2. Per-target Z-scores for FEIG-S (013), Bhattacharya-Server (149), Seok-server (070), MULTICOM-CLUSTER (075), and MUFOLD (081) in CASP14 in terms of GDT-HA, RMSD, GDC-sc, SphereGrinder, and MolProbity scores as well as per-target overall Z-score calculated as the weighted sum of Z-scores for GDT-HA, GDC-sc, RMSD, SphereGrinder, and MolProbity.

Supporting Figures

Figure S1. Interactive quantitative and visual analysis of the refinement results by DeepRefiner.

Figure S2. Degree of refinement for “Bhattacharya-Server” considering all CASP13 and CASP14 refinement targets grouped by various length bins. Distributions of ΔGDT-HA, ΔGDC-sc, and ΔMolProbilty scores for various length bins considering the best submission presented. Regions shaded in black illustrate improvement over the starting structure. Numbers above the distribution plots indicate the percentages of refinement successes and failures.

Figure S3. Degree of refinement for “Bhattacharya-Server” considering all CASP13 and CASP14 refinement targets grouped by various starting GDT-HA bins. Distributions of ΔGDT-HA, ΔGDC-sc, and ΔMolProbilty scores for various starting GDT-HA bins considering the best submission presented. Regions shaded in black illustrate improvement over the starting structure. Numbers above the distribution plots indicate the percentages of refinement successes and failures.

Supporting Text

Text S1. Feature generation for the advanced error estimation module of DeepRefiner.

The advanced error estimation module of DeepRefiner based on deep residual neural networks (ResNet) uses both sequence-based predicted and structural features as well as the features based on the consistency between predicted and observed structural properties to represent each of the residues in the structure. The overall feature generation strategy is briefly described below:

Generation of Multiple Sequence Alignment (MSA): To generate Multiple Sequence Alignment (MSA), we perform three iterations of HHblits (1) search with an E-value inclusion of 10^{-3} against the protein sequence database Uniclust30 (2) with a query sequence coverage of 10% and pairwise sequence identity of 90%. Subsequently, the generated MSA is used for predicting various sequence-based features including secondary structure, solvent accessibility, and inter-residue distance maps.

Generation of Position-Specific Scoring Matrix (PSSM)**:** We generate PSSM from the query sequence by performing a search against the NR database using PSI-BLAST v2.2.26 software (3) with an E-value of 0.001.

Prediction of secondary structure, solvent accessible surface area, and backbone dihedral angles: We use SPIDER3 (4) to predict three-class secondary structures (H/E/C), solvent accessible surface area, and backbone dihedral angles (ϕ, ψ) from the query sequence. We extract the observed secondary structure and solvent accessible surface area using DSSP (5) or STRIDE (6).

Prediction of inter-residue distance maps: We use DMPfold (7) to predict the inter-residue distance maps. We obtain the rawdistpred.current file generated by DMPfold as the initial prediction without any iterative refinement. For each of the interacting residue pairs, the distance bins and their associated likelihoods are subsequently used for generating distance-based features.

Featurization: We use 23 features for each residue in the structures that include:

(i) Sequence profile conservation score: We extract information per-position score for each residue in the starting structure from the generated PSSM and subsequently apply the sigmoid function to normalize the scores between 0 and 1 to be used as a feature.

(ii) The number of effective sequences: We use calNf_ly program (8) that accepts Multiple Sequence Alignment (MSA) to calculate the number of effective sequences (NEFF) by summing up the nth reciprocal of the total number of sequences in the MSA with sequence identity greater than 80% to the corresponding sequence. The resulting NEFF is used as a feature.

(iii) Agreement between predicted and true secondary structure and solvent accessibility: We calculate the residue-specific sequence-structure agreement between the predicted and observed secondary structure and solvent accessibility. For each of the residues in the structure, we assign a feature value of

1 if both predicted and observed secondary structures agree and 0 otherwise. Furthermore, we calculate the squared error between the predicted and observed solvent accessibility and subsequently apply the sigmoidal transformation to normalize the squared error. We then use them as two features.

(iv) Angular RMSD: We calculate backbone dihedral angles (ϕ, ψ) for each of the residues in the structure. Afterwards, we calculate the angular root mean square deviation between the observed dihedral angles (ϕ_o, ψ_o) and SPIDER3 (4) predicted backbone dihedral angles (ϕ_p, ψ_p) as follows:

$$Angular\ RMSD = \sqrt{\frac{1}{n}\sum_i(\min(|x_{oi} - x_{pi}|, 2\pi - |x_{oi} - x_{pi}|)^2}$$

$$Normalized\ Angular\ RMSD = \frac{1}{1 + \left(\frac{Angular\ RMSD}{\pi/4}\right)^2}$$

Where x_o and x_p represent the vector of observed and predicted ϕ or ψ dihedral angles for n residues in the structure. We subsequently normalize the angular RMSD values and use them as two features.

(v) Distance-based weighted histogram alignment score: We use DMPfold predicted distance histogram (i.e., rawdistpred.current file) to extract inter-residue distance distribution at 5 evenly distributed distance intervals of 6, 8, 10, 12, and 14Å by summing up the likelihoods values of all the bins below a specific distance threshold. To minimize noise, we discard all residue pairs with likelihood values below 0.2. We also calculate the observed distance histogram at 6, 8, 10, 12, and 14Å from the structure to perform eigen-decomposition and dynamic programming-based alignment between the predicted and observed histogram resulting in 5 distance alignment scores. We subsequently multiply the raw alignment scores at 6, 8, 10, 12, and 14Å with empirically selected weights of 0.10, 0.25, 0.30, 0.25, and 0.10, respectively, and use them as 5 distance-based features.

(vi) Rosetta centroid energy terms: We calculate 12 *Rosetta* centroid energy terms (9, 10) including the residue environment (env), residue pair interactions (pair), cβ density (cbeta), steric repulsion (vdw), radius of gyration (rg), packing (cenpack) density, contact order (co), statistical potentials for secondary structure formation (hs_pair, ss_pair, sheet, rsigma), and centroid hydrogen bonding (cen_hb). Once again, we use the sigmoid function to normalize the scores between 0 and 1 before using as features.

Text S2. Training an ensemble of deep residual neural networks at fine-grained error thresholds.

The advanced version of residue-level error estimator used in DeepRefiner is an improvement over the deep networks utilized in our original refinement protocol refineD (11). The advanced error estimation module of DeepRefiner employs an ensemble of deep residual neural networks (ResNet) trained at finer-grained distance thresholds following a similar strategy as leveraged by our successful application of protein model quality estimation method QDeep (12). Specifically, each ResNet classifier consists of 13 residual blocks, each consisting of three 1D convolutional layers with a kernel size of 1 × 1, 1 × 3, and 1 ×

1, which is similar to the bottleneck design (13). Each 1D convolutional neural network layer uses an L2-norm regularization with a weight decay parameter of 0.0001 to prevent overfitting (14). 1 × 1 layers, also known as "bottleneck layers" at the end of each residual block are used to preserve the dimension of the network by reducing and restoring the dimensionality of the input feature vector. Each classifier takes an input with a shape of L × 483 × 1 to its first convolutional layer with a kernel and filter size of 1 × 7 and 64 × 1, which is subsequently transformed into a 64-dimensional feature vector using 1 × 1 convolutional layer of the first residual block with a filter size of 64 × 1. Each residual block in the network also establishes a "shortcut or skip connection" between the input and the output layer, performing as an identity mapping between the input and the output layer. We adopt a multi-stage design of the ResNet model by sequential stacking 3 consecutive stages having 3, 4, and 6 residual blocks in the first, second, and third stage, respectively, with an output channel dimension of 128, 256, and 512, respectively. At the end of the residual blocks, we use an average pooling layer with a pool size of 2 that helps in faster computation by halving the number of parameters. Afterwards, we add a flatten layer that accepts the pooled features and transformed them into a 1D vector. The fully connected (dense layer) layer at the end accepts the 1D vector and applies sigmoidal activation to return a probability value for residue-level error classification.

To perform residue-level ensemble error classification, we independently train four binary classifiers that can estimate the residue-level C_α errors at four error thresholds of 0.5, 1, 2, and 4Å, analogous to GDT-HA. The ensemble classifiers leverage the same features for representing each residue in the structures as described before. We assign a binary label to each residue of the starting structure by calculating the Euclidian distance error between the C_α atom of a residue and the corresponding aligned residue in the experimental structure after performing the sequence-dependent analysis between the model and the corresponding experimental structure using the LGA program (15). While assigning labels, we consider four fine-grained error thresholds of 0.5, 1, 2, and 4Å and assign a label of 1 if the distance error is within a threshold and 0 otherwise. We train the ensemble classifiers by using a maximum number of epochs of 120 and an optimal batch size of 64 that best fits the GPU limit. To avoid overfitting, we use EarlyStopping callback of Keras (16) with a patience value of 20. We optimize the model using the binary crossentropy loss function and first-order gradient-based Adam optimizer. After training, each binary classifier estimates the residue-level errors at four fine-grained thresholds of 0.5, 1, 2, and 4Å. Collectively, the set of four classifiers results in residue-level ensemble error classifications.

Text S3. Iterative restrained relaxation for guiding protein structure refinement.

After performing residue-level ensemble error classifications, we subsequently convert the error estimates into multi-resolution probabilistic restraints weighted by their associated likelihood values and apply on the C_α atom of the starting structure in the form of Rosetta Coordinate Constraint with FLAT_HARMONIC function at 0.5, 1, 2, and 4Å thresholds in conjunction with the Rosetta's full atom Energy Function (17) to

perform restrained relaxation. We use Rosetta's FastRelax application (18, 19), which inherently performs several rounds of side-chain repacking and gradient-based backbone minimization. We generate a total of 100 refined models by iteratively applying restrained relaxation. Restraints are imposed simultaneously in a cumulative manner for conservative refinement mode aimed at achieving consistently positive refinement, whereas imposing restraints in a non-cumulative manner independent of each other leads to adventurous refinement mode aimed at producing higher degree of structural changes.

Text S4. Global and local quality estimation of the refined structures.

DeepRefiner uses the residue-level ensemble error classifications to estimate the global and local quality of the refined structures. Ensemble of DeeCNF and ResNet classifiers estimate the likelihood of each residue in the structure to be within rÅ (r ∈ {0.5, 1, 2, 4} Å) threshold. A likelihood cutoff of 0.5 is then used for classifying a residue as 1 and 0 otherwise. Subsequently, the global quality score of a refined structure is calculated analogous to GDT-HA by a weighted combination of the residue-level ensemble error classifications at 0.5, 1, 2, and 4Å thresholds as follows:

$$global\_quality = \frac{N_{0.5} + N_1 + N_2 + N_4}{4 \times L}$$

Where L is the length of the structure and $N_{0.5}$, N_1, N_2, and N_4 are the number of residues predicted to be aligned with their corresponding residues in the native structure at 0.5, 1, 2, and 4Å thresholds. Thus, *global_quality* score ranges between [0, 1], with a higher score indicating better global quality.

DeepRefiner further uses the residue-level error estimates at 0.5, 1, 2, and 4Å thresholds to estimate the residue-level quality score by employing an inverse S-score calculation as follows:

$$local\_quality_i(r) = \frac{\sum_{r \in \{0.5,1,2,4\}} \left(\frac{r \times \left(\sqrt{1 - p_r}\right)}{p_r} \right)}{4}$$

Where p_r denotes the probability of ith residue in the structure, predicted by a specific classifier at rÅ (r ∈ {0.5, 1, 2, 4} Å) threshold.

Text S5: Refinement evaluation.

To evaluate the performance of "Bhattacharya-Server" participating as a server group in CASP13 and CASP14 refinement category, we use a combination of several accuracy measures including GDT-HA (20), RMSD (21), GDC-sc (22), SphereGrinder (23), and MolProbity (24). Specifically, we calculate the Z-scores of various accuracy measures as routinely performed by CASP assessors (25) by following a two-step procedures considering all top-ranked submissions. In the first step, all submissions with a Z-score lower than -2 are discarded. In the second step, we use a penalty threshold of 0 in calculating the final Z-

score using the scores obtained from the first step. The penalty threshold of 0 assigns a score of 0 to any model having a Z-score less than 0. We subsequently rank the participating server groups based on the sum of overall Z-score. Following previous CASP refinement assessment (26), the overall Z-score is calculated as the weighted sum of Z-scores of GDT-HA, RMSD, GDC-sc, SphereGrinder, and MolProbity scores as follows:

$$Overall\ Z - score = \frac{4 \times Z_{GDT\_HA} - Z_{rmsd} + Z_{GDC-sc} + Z_{sphGr} - Z_{MP}}{8}$$

The above scoring scheme emphasizes the accuracy of backbone positioning as measured by GDT-HA score, which is widely used by the CASP assessors for refinement evaluation, while integrating other complementary aspects of refinement including side-chain quality, stereochemistry, and physical realism. The per-target Z-scores in terms of GDT-HA, RMSD, GDC-sc, SphereGrinder, and MolProbity scores as well as the overall Z-score calculated as the weighted sum of Z-scores for top server groups participating in CASP13 and CASP14 refinement experiments are reported in **Table S1** and **Table S2**, respectively.

Supporting Tables

Table S1. Per-target Z-scores for Seok-server (156), Bhattacharya-Server (102), YASARA (004), MUFold_server (312), and 3DCNN (359) in CASP13 in terms of GDT-HA, RMSD, GDC-sc, SphereGrinder and MolProbity scores as well as per-target overall Z-score calculated as the weighted sum of Z-scores for GDT-HA, GDC-sc, RMSD, SphereGrinder, and MolProbity.

Target	GDT-HA					RMSD					GDC-sc					SphereGrinder					MolProbity					Overall				
	156	102	004	312	359	156	102	004	312	359	156	102	004	312	359	156	102	004	312	359	156	102	004	312	359	156	102	004	312	359
R0957s2	0.467	1.169	0.752	0.000	0.000	-0.314	-0.765	-0.177	0.000	0.000	0.757	1.195	1.007	0.000	0.000	0.812	0.539	0.110	0.000	0.000	-1.008	-0.390	-0.775	0.000	0.000	0.595	0.946	0.635	0.000	0.000
R0949	1.327	0.049	0.000	0.401	0.000	-0.658	-0.181	0.000	-1.107	0.000	1.425	0.224	0.000	0.837	0.000	0.651	0.110	0.000	1.355	0.000	-0.906	-0.556	0.000	0.000	0.000	1.118	0.158	0.000	0.613	0.000
R0959	0.304	0.216	0.000	0.188	0.000	-0.619	-0.082	0.000	-0.171	0.000	0.532	0.313	0.360	0.118	0.000	0.019	0.208	0.208	0.525	0.000	-1.257	-0.593	-0.876	0.000	0.000	0.455	0.258	0.180	0.196	0.000
R0962	1.079	0.000	0.000	0.499	0.000	0.000	0.000	-0.501	-0.695	0.000	0.913	0.000	0.030	0.259	0.000	0.000	0.000	0.000	1.354	0.000	-1.073	-0.404	-0.910	0.000	0.000	0.788	0.051	0.180	0.538	0.000
R0968s1	0.381	0.000	0.202	0.052	0.000	-0.556	-0.216	-0.492	0.000	0.000	0.540	0.000	0.010	0.000	0.000	1.045	0.309	0.677	0.217	0.000	-1.094	-1.094	-1.094	0.000	0.000	0.595	0.202	0.385	0.053	0.000
R0968s2	0.000	0.362	0.891	0.000	0.000	-0.843	-0.052	-0.198	0.000	0.000	0.132	0.702	0.862	0.156	0.000	0.244	0.017	0.921	0.017	0.000	-0.767	-0.535	-1.505	0.000	0.000	0.248	0.344	0.881	0.022	0.000
R0974s1	0.496	0.119	0.684	0.382	0.000	-1.097	0.000	0.000	0.000	0.000	0.792	0.000	0.774	0.148	0.000	1.336	0.214	0.214	0.000	0.088	-0.908	-0.908	-0.708	0.000	0.000	0.764	0.200	0.554	0.210	0.011
R0976-D1	1.109	0.999	0.700	0.347	0.000	-1.133	-0.288	-0.417	-0.717	0.000	1.288	1.240	0.596	0.000	0.000	2.005	0.070	0.220	1.112	0.000	-0.925	-0.353	-1.393	0.000	0.000	1.223	0.743	0.678	0.402	0.000
R0976-D2	1.271	0.839	1.175	0.335	0.000	0.000	-1.007	-0.862	-0.131	0.000	1.284	1.364	0.706	0.378	0.000	0.586	0.455	0.542	0.237	0.000	-0.947	-0.751	-1.296	0.000	0.000	0.988	0.867	1.013	0.261	0.000
R0977-D2	0.884	0.487	0.054	0.106	0.000	-0.102	-0.679	-0.871	0.000	-0.132	0.718	1.024	0.306	0.063	0.000	1.068	0.764	0.579	0.000	0.000	-1.252	-0.390	-1.032	0.000	0.000	0.835	0.600	0.375	0.061	0.016
R0979	0.314	1.415	0.754	0.000	0.000	0.000	-0.745	-0.559	0.000	-0.192	2.004	0.000	0.217	0.000	1.200	0.266	0.000	0.266	0.000	0.000	-1.116	-0.320	-0.320	0.000	0.000	0.580	0.840	0.547	0.000	0.174
R0981-D3	1.229	0.000	0.000	0.589	0.000	-0.794	-0.557	-0.609	0.000	-0.497	1.142	0.556	0.929	0.789	0.000	0.828	0.000	0.000	1.504	0.000	-0.622	-0.295	-1.399	0.000	0.000	1.038	0.176	0.367	0.581	0.062
R0981-D4	0.784	0.848	0.000	0.000	0.000	-0.232	0.000	0.000	0.000	-0.621	0.645	0.871	0.000	0.000	0.114	0.065	0.598	0.000	0.000	0.065	-0.877	-0.268	-1.578	0.000	-0.176	0.619	0.641	0.197	0.000	0.122
R0981-D5	1.696	0.389	0.000	0.000	0.000	-1.957	0.000	0.000	0.000	0.000	0.210	0.969	0.452	0.000	0.000	0.648	0.000	0.000	0.000	0.000	-1.088	-0.745	-1.088	0.000	-0.059	1.336	0.409	0.192	0.000	0.007
R0982-D2	1.074	0.000	0.000	0.643	0.000	-0.721	-0.255	0.000	-0.101	0.000	1.118	0.371	0.271	0.372	0.000	0.698	0.492	0.289	0.903	0.000	-0.849	-0.657	-1.564	0.000	0.000	0.961	0.222	0.265	0.494	0.000
R0986s1	0.585	0.585	0.230	0.000	0.000	-0.505	-0.588	-0.408	0.000	0.000	0.443	0.842	0.195	0.000	0.000	0.748	0.678	0.609	0.000	0.260	-1.134	-0.883	-1.134	0.000	0.000	0.646	0.666	0.408	0.000	0.032
R0986s2	0.000	0.006	0.704	0.418	0.000	0.000	-0.426	-0.586	-0.693	-0.100	0.251	0.000	0.327	0.384	0.000	0.000	0.314	0.693	0.807	0.000	-0.535	-0.505	-1.703	0.000	0.000	0.098	0.158	0.766	0.445	0.012
R0989-D1	1.327	0.409	1.098	0.000	0.000	-0.852	-0.680	-0.757	0.000	-0.773	1.808	0.299	1.324	0.000	0.000	1.075	0.753	0.753	0.000	0.000	-0.892	-0.425	-1.428	0.000	0.000	1.242	0.474	1.082	0.000	0.097
R0992	1.877	0.887	0.889	0.591	0.000	-0.480	-0.066	-0.216	-1.079	-0.326	1.486	0.893	0.725	0.000	0.000	1.371	0.391	0.064	0.636	0.000	-1.147	-0.826	-1.244	0.000	0.000	1.499	0.716	0.726	0.510	0.041
R0993s2	0.426	0.702	0.000	0.426	0.000	-0.367	-1.037	-0.595	0.000	0.000	1.956	1.755	0.000	0.898	0.000	1.402	0.141	0.897	0.000	0.000	-1.200	-0.536	-1.549	0.000	0.000	0.829	0.784	0.380	0.325	0.000
R0996-D4	0.249	0.000	0.249	2.278	0.000	-1.126	0.000	0.000	-2.585	0.000	0.092	0.000	0.239	1.022	0.000	0.055	0.000	0.217	2.086	0.000	-0.943	-0.447	-1.680	0.000	0.000	0.402	0.056	0.392	1.851	0.000
R0996-D5	0.209	0.362	0.093	0.903	0.000	-0.232	0.000	-0.239	-0.402	0.000	0.040	0.519	1.092	0.092	0.000	0.000	0.000	0.690	1.536	0.000	-0.956	-0.878	-0.784	0.000	0.000	0.258	0.355	0.397	0.705	0.000
R0996-D7	0.739	0.010	0.321	0.807	0.000	-0.126	-0.090	-0.401	-0.283	-0.234	0.889	1.458	0.832	0.536	0.000	1.492	0.265	0.469	0.265	0.000	-0.977	-0.802	-1.036	0.000	-0.140	0.805	0.332	0.503	0.539	0.047
R0997	0.824	0.022	0.000	0.978	0.000	-0.905	-0.772	-0.369	-0.857	-0.426	0.000	0.581	0.056	0.299	0.000	0.528	0.875	0.355	1.172	0.207	-1.233	-0.581	-1.208	0.000	0.000	0.745	0.362	0.248	0.780	0.079
R0999-D3	0.708	0.763	0.140	0.980	0.000	-1.023	-0.763	-0.525	-0.861	0.000	0.926	0.836	0.740	0.658	0.000	0.916	0.562	0.613	0.463	0.000	-0.868	-0.383	-1.484	0.000	0.000	0.821	0.700	0.490	0.738	0.000
R1001	0.244	0.592	0.000	0.322	0.000	0.000	0.000	0.000	-0.626	0.000	0.000	0.257	0.000	0.830	0.000	0.000	0.000	0.682	0.818	0.000	-0.621	-0.497	-1.435	0.000	0.000	0.200	0.390	0.265	0.445	0.000
R1002-D2	1.222	0.387	0.596	0.701	0.000	-0.324	-0.357	0.000	-0.613	0.000	0.889	0.242	0.589	0.363	0.000	0.471	0.347	0.000	0.595	0.000	-1.196	-0.316	-1.057	0.000	0.000	0.971	0.351	0.504	0.547	0.000
R1004-D2	0.000	0.490	0.000	0.000	0.000	-0.024	-0.420	0.000	0.000	0.000	0.619	0.142	0.460	0.000	0.000	0.651	0.859	0.000	0.336	0.000	-1.175	-1.175	-1.143	0.000	0.000	0.309	0.570	0.200	0.042	0.000
R1016	0.699	0.676	0.000	0.845	0.000	-0.411	0.000	0.000	0.000	0.000	0.423	0.486	0.204	0.178	0.000	0.957	0.704	0.000	0.766	0.000	-1.157	-0.543	-1.102	0.000	0.000	0.718	0.555	0.163	0.540	0.000
**Sum**	21.526	12.780	9.533	12.790	0.000	-15.402	-10.026	-8.781	-10.923	-3.302	23.323	17.139	13.303	8.381	1.314	19.934	9.665	10.067	16.702	0.620	-28.724	-17.056	-33.524	0.000	-0.374	21.686	13.125	12.976	10.895	0.701

Table S2. Per-target Z-scores for FEIG-S (013), Bhattacharya-Server (149), Seok-server (070), MULTICOM-CLUSTER (075), and MUFOLD (081) in CASP14 in terms of GDT-HA, RMSD, GDC-sc, SphereGrinder and MolProbity scores as well as per-target overall Z-score calculated as the weighted sum of Z-scores for GDT-HA, GDC-sc, RMSD, SphereGrinder, and MolProbity.

Target	GDT-HA					RMSD					GDC-sc					SphereGrinder					MolProbity					Overall				
	013	149	070	075	081	013	149	070	075	081	013	149	070	075	081	013	149	070	075	081	013	149	070	075	081	013	149	070	075	081
R1029	0.123	0.396	0.805	0.668	0.000	-0.489	-0.358	-0.717	-0.362	0.000	0.131	0.670	0.512	0.577	0.000	0.000	0.870	0.378	1.198	0.000	-1.191	0.000	-1.023	0.000	0.000	0.288	0.435	0.731	0.601	0.000
R1029x1	0.135	0.435	0.000	0.736	0.000	-0.246	-0.363	0.000	-0.458	-0.033	0.000	0.531	0.000	0.579	0.000	0.000	0.942	0.000	1.276	0.000	-0.793	0.000	0.000	0.000	0.000	0.197	0.447	0.000	0.657	0.004
R1030-D2	1.426	0.000	0.000	0.000	0.000	-0.891	-0.296	-0.152	-0.247	0.000	1.297	0.000	0.000	0.000	0.000	1.644	0.574	0.000	0.306	0.000	-0.930	-0.930	-0.930	-0.081	0.000	1.309	0.225	0.135	0.079	0.000
R1031	0.846	0.332	0.290	0.076	0.000	-0.663	-0.519	-0.441	-0.535	0.000	0.961	0.552	0.324	0.000	0.000	0.894	0.112	0.242	0.372	0.000	-1.373	-0.948	-0.899	-0.049	0.000	0.909	0.432	0.383	0.158	0.000
R1033	0.000	0.435	0.397	0.630	0.000	-0.382	-0.621	-0.501	-0.600	0.000	0.000	1.106	0.951	0.748	0.000	0.132	0.132	1.742	0.416	0.000	-0.870	0.000	-1.276	0.000	0.000	0.173	0.450	0.757	0.535	0.000
R1034	0.692	0.841	1.213	0.395	0.000	-0.261	-0.514	-0.141	-0.609	-1.438	1.123	0.925	0.436	0.681	0.000	0.222	0.742	0.352	0.872	0.482	-1.160	-1.112	-1.052	0.000	0.000	0.692	0.832	0.854	0.468	0.240
R1034x1	1.120	0.642	0.000	0.695	0.749	-0.976	-0.689	0.000	-0.778	0.000	0.720	0.582	0.000	0.662	0.000	0.575	0.890	0.000	0.812	0.100	-1.094	-1.193	0.000	0.000	0.000	0.981	0.741	0.000	0.629	0.387
R1035	0.963	0.690	0.878	0.816	0.000	-0.874	-0.731	-0.033	-0.745	0.000	0.758	0.661	1.282	0.602	0.000	0.728	0.633	0.000	0.775	0.000	-1.119	-0.894	-0.906	0.000	0.000	0.916	0.710	0.717	0.673	0.000
R1038-D2	0.687	0.160	0.495	0.447	0.000	-0.492	-0.400	-0.348	-0.146	-0.210	1.010	0.751	0.310	0.129	0.000	0.263	1.026	0.167	0.454	0.000	-1.208	-0.369	-0.966	0.000	0.000	0.715	0.398	0.471	0.315	0.026
R1039	1.551	0.000	0.247	0.147	0.000	-0.644	-0.437	-0.542	-0.455	0.000	1.924	0.187	0.602	0.059	0.000	0.873	0.420	0.470	0.320	0.000	-1.130	-0.168	-0.563	0.000	0.000	1.347	0.151	0.396	0.178	0.000
R1040v1	0.436	0.601	0.471	0.000	0.000	-0.746	-0.816	-0.064	0.000	0.000	0.164	0.823	0.789	0.000	0.000	0.658	0.709	0.081	0.000	0.000	-0.837	-0.401	-0.837	0.000	0.000	0.519	0.644	0.457	0.000	0.000
R1040v2	1.682	0.000	0.523	0.000	0.000	-0.656	-0.414	-0.364	0.000	0.000	1.615	0.000	0.050	0.000	0.000	0.860	1.095	0.623	0.000	0.000	-0.989	-0.170	-0.905	0.000	0.000	1.356	0.210	0.504	0.000	0.000
R1041v1	0.000	0.835	0.491	0.000	1.006	-0.571	-0.704	-0.371	0.000	-0.724	0.000	1.039	0.594	0.000	0.488	0.476	0.631	0.553	0.000	0.682	-0.867	-0.615	-1.035	0.000	0.000	0.239	0.791	0.565	0.000	0.739
R1041v2	1.732	0.134	0.220	0.000	0.000	-1.095	-0.578	-0.617	0.000	0.000	1.614	0.218	0.091	0.000	0.037	0.511	0.706	1.011	0.000	0.844	-1.216	-0.762	-1.037	0.000	0.000	1.421	0.350	0.455	0.000	0.110
R1042v1	0.000	0.648	0.226	0.000	0.000	-0.336	-0.070	-0.864	0.000	0.000	0.000	1.017	0.893	0.000	0.000	0.000	1.310	0.000	0.000	0.000	-0.857	-0.702	-0.952	0.000	0.000	0.149	0.711	0.452	0.000	0.000
R1042v2	0.000	0.819	0.793	0.000	0.000	-0.420	-0.813	0.000	0.000	0.000	0.000	1.130	0.901	0.000	0.000	0.358	0.900	0.000	0.000	0.000	-1.156	-0.923	-0.923	0.000	0.000	0.242	0.880	0.624	0.000	0.000
R1043v1	1.857	0.117	0.355	0.000	0.000	-0.707	-0.479	-0.584	0.000	0.000	0.721	0.231	0.741	0.000	0.000	1.166	0.575	1.209	0.000	0.000	-1.195	-0.591	-0.712	0.000	0.000	1.402	0.293	0.583	0.000	0.000
R1043v2	0.794	0.385	0.361	0.000	0.000	-0.478	-0.585	-0.450	0.000	0.000	0.367	1.191	0.478	0.000	0.000	0.332	0.597	0.755	0.000	0.000	-0.651	-0.971	-0.971	0.000	0.000	0.626	0.610	0.512	0.000	0.000
R1045s2	0.000	0.460	0.395	0.633	0.000	-0.144	-0.097	0.000	-0.130	0.000	0.373	0.414	0.097	0.530	0.000	0.973	0.339	0.000	0.402	0.339	-0.930	-0.694	-0.773	0.000	0.000	0.302	0.423	0.306	0.449	0.042
R1049	0.365	0.541	0.365	0.507	0.000	0.000	-0.787	0.000	-0.503	0.000	0.145	0.219	0.373	0.436	0.000	0.000	0.591	0.527	0.653	0.151	-1.144	-0.711	-0.521	0.000	0.000	0.344	0.559	0.360	0.452	0.019
R1052-D2	0.313	1.150	1.049	0.746	0.285	-0.579	-0.693	-0.613	-0.674	-0.347	0.507	0.976	0.846	0.887	0.101	0.921	0.351	0.351	0.600	0.243	-1.017	-0.418	-0.902	0.000	0.000	0.535	0.880	0.863	0.643	0.229
R1053v1	1.967	0.000	0.410	0.000	0.000	-0.938	-0.755	-0.802	0.000	-0.331	1.964	0.024	0.590	0.000	0.000	0.873	0.714	0.792	0.000	0.240	-1.233	-0.982	-1.233	0.000	0.000	1.610	0.309	0.632	0.000	0.071
R1053v2	0.344	0.762	0.000	0.000	0.000	-0.838	-0.951	0.000	0.000	0.000	0.660	0.977	0.260	0.000	0.000	0.459	0.690	0.000	0.000	0.000	-0.978	-0.978	-0.882	0.000	0.000	0.539	0.831	0.143	0.000	0.000
R1055	0.707	0.292	0.754	0.844	0.064	0.000	0.000	-1.796	0.000	0.000	0.000	0.428	0.531	0.341	0.000	0.000	0.107	0.903	0.000	0.000	-0.830	-0.776	-1.165	0.000	0.000	0.457	0.310	0.926	0.465	0.032
R1055x1	0.656	0.582	0.000	0.914	0.766	0.000	0.000	0.000	-0.194	-0.143	0.321	0.590	0.000	0.471	0.013	0.000	0.000	0.000	0.070	0.000	-1.182	-1.182	0.000	0.000	0.000	0.516	0.513	0.000	0.549	0.403
R1056	1.839	0.549	0.771	0.697	0.000	0.000	-0.389	-0.350	-0.367	-0.607	0.658	0.890	0.280	0.536	0.000	0.355	1.100	0.761	0.830	0.000	-1.282	-0.284	-0.801	0.000	0.000	1.207	0.608	0.660	0.565	0.076
R1056x1	1.662	0.796	0.000	0.884	0.000	-0.166	-0.489	0.000	-0.419	-0.361	1.363	1.490	0.000	0.593	0.000	0.393	1.240	0.000	0.937	0.000	-1.299	-0.501	0.000	0.000	0.000	1.234	0.863	0.000	0.686	0.045
R1057	1.762	0.314	0.255	0.080	0.285	-0.274	-0.591	0.000	-0.683	-0.979	1.096	0.589	0.304	0.596	0.302	0.415	0.646	0.000	0.703	0.241	-1.459	-1.034	-0.888	0.000	0.000	1.287	0.514	0.277	0.288	0.333
R1061-D3	2.239	0.422	0.908	0.000	0.000	-1.324	-0.431	0.000	-0.468	0.000	1.550	0.375	1.012	0.384	0.000	0.726	0.825	0.974	0.726	0.000	-1.013	0.000	-0.869	0.000	0.000	1.696	0.415	0.811	0.197	0.000
R1065s1	1.082	0.407	0.436	0.660	0.000	-0.766	-0.706	0.000	-0.778	0.000	1.271	0.588	0.578	1.036	0.000	0.505	0.255	0.380	0.629	0.000	-0.991	-0.600	-0.991	0.000	0.000	0.983	0.472	0.461	0.636	0.000
R1065s2	0.875	0.749	0.000	0.464	0.000	-0.670	-0.708	0.000	-0.600	-0.431	0.713	0.993	0.194	0.668	0.000	0.034	0.651	0.000	0.651	0.000	-1.060	-0.606	-0.929	0.000	0.000	0.747	0.744	0.140	0.472	0.054
R1067v1	0.297	0.362	0.233	0.000	0.000	0.000	0.000	-0.271	0.000	-0.796	0.384	0.183	0.101	0.000	0.000	0.111	0.348	0.071	0.000	0.000	-0.785	-0.702	-0.523	0.000	0.000	0.309	0.335	0.237	0.000	0.100
R1067v2	0.491	0.716	0.267	0.000	0.000	-0.503	-0.570	0.000	0.000	0.000	0.534	0.973	0.402	0.000	0.000	0.396	0.600	0.151	0.000	0.000	-1.229	-1.027	-0.889	0.000	0.000	0.578	0.754	0.314	0.000	0.000
R1067x1	0.498	0.361	0.000	0.596	0.361	0.000	0.000	0.000	0.000	0.000	0.040	0.228	0.000	0.000	0.000	0.000	0.085	0.000	0.193	0.230	-1.133	-0.963	0.000	0.000	0.000	0.395	0.340	0.000	0.322	0.209
R1068	1.201	0.558	0.399	0.622	0.365	-0.562	-0.406	-0.336	-0.425	-0.360	0.844	0.085	0.404	0.472	0.000	0.918	0.502	0.000	1.084	0.668	-0.861	-0.798	-1.292	0.000	0.000	0.999	0.503	0.454	0.559	0.311
R1068x1	1.648	0.416	0.000	0.563	0.681	-0.775	-0.351	0.000	-0.363	-0.374	1.428	0.455	0.000	0.320	0.000	0.439	0.605	0.000	1.020	1.020	-1.081	-1.244	0.000	0.000	0.000	1.290	0.540	0.000	0.494	0.515
R1074v1	0.381	0.338	0.467	0.000	0.000	-0.358	-0.717	0.000	0.000	0.000	0.458	0.609	0.362	0.000	0.000	0.412	0.815	0.000	0.000	0.000	-1.026	-0.779	-0.826	0.000	0.000	0.472	0.534	0.382	0.000	0.000
R1074v2	0.000	0.000	0.000	0.000	0.000	-0.048	-0.089	-0.033	0.000	0.000	0.000	0.000	0.000	0.000	0.000	0.073	0.036	0.000	0.000	0.000	-0.896	-0.495	-0.953	0.000	0.000	0.127	0.078	0.123	0.000	0.000

R1074x2	0.083	0.000	0.000	0.000	0.000	-0.265	-0.062	0.000	-0.304	0.000	0.039	0.000	0.000	0.000	0.000	0.333	0.123	0.000	0.123	0.000	-1.030	-0.494	0.000	0.000	0.000	0.250	0.085	0.000	0.053	0.000
R1078	1.884	0.569	0.737	0.428	0.233	-0.627	-0.829	0.000	-0.849	0.000	1.896	0.746	0.458	0.621	0.000	1.246	0.504	0.391	0.904	0.391	-1.045	-0.709	-0.733	0.000	0.000	1.544	0.633	0.566	0.511	0.166
R1082	0.198	0.327	1.420	0.135	0.000	-0.671	-0.396	-1.223	-0.373	0.000	0.665	0.239	1.213	0.000	0.000	0.149	0.772	0.875	0.564	0.000	-0.766	-0.741	-0.221	0.000	0.000	0.380	0.432	1.151	0.184	0.000
R1085-D1	0.930	0.000	0.000	0.000	0.000	-0.635	0.000	-0.111	0.000	0.000	1.234	0.000	0.000	0.000	0.000	0.945	0.306	0.227	0.227	0.546	-1.224	-0.414	-0.862	0.000	0.000	0.970	0.090	0.150	0.028	0.068
R1090	2.083	0.240	0.326	0.050	0.000	-0.770	-0.411	-0.683	-0.393	0.000	2.062	0.546	0.308	0.142	0.000	2.271	0.863	0.416	0.353	0.000	-1.043	-0.870	-1.109	0.000	0.000	1.810	0.456	0.478	0.136	0.000
R1091-D2	1.525	0.000	0.172	0.250	0.000	-1.129	-0.689	-0.617	-0.664	0.000	1.203	0.384	0.394	0.345	0.000	0.969	0.623	0.574	0.623	0.000	-0.879	-0.629	-0.704	0.000	0.000	1.285	0.291	0.372	0.329	0.000
**Sum**	**39.077**	**18.381**	**17.128**	**13.684**	**4.793**	**-22.970**	**-20.506**	**-13.022**	**-13.120**	**-7.134**	**33.815**	**24.612**	**17.662**	**12.415**	**0.942**	**23.608**	**26.555**	**14.977**	**18.096**	**6.178**	**-46.051**	**-29.380**	**-33.056**	**-0.130**	**0.000**	**35.344**	**21.822**	**18.404**	**12.312**	**4.178**

Supporting Figures

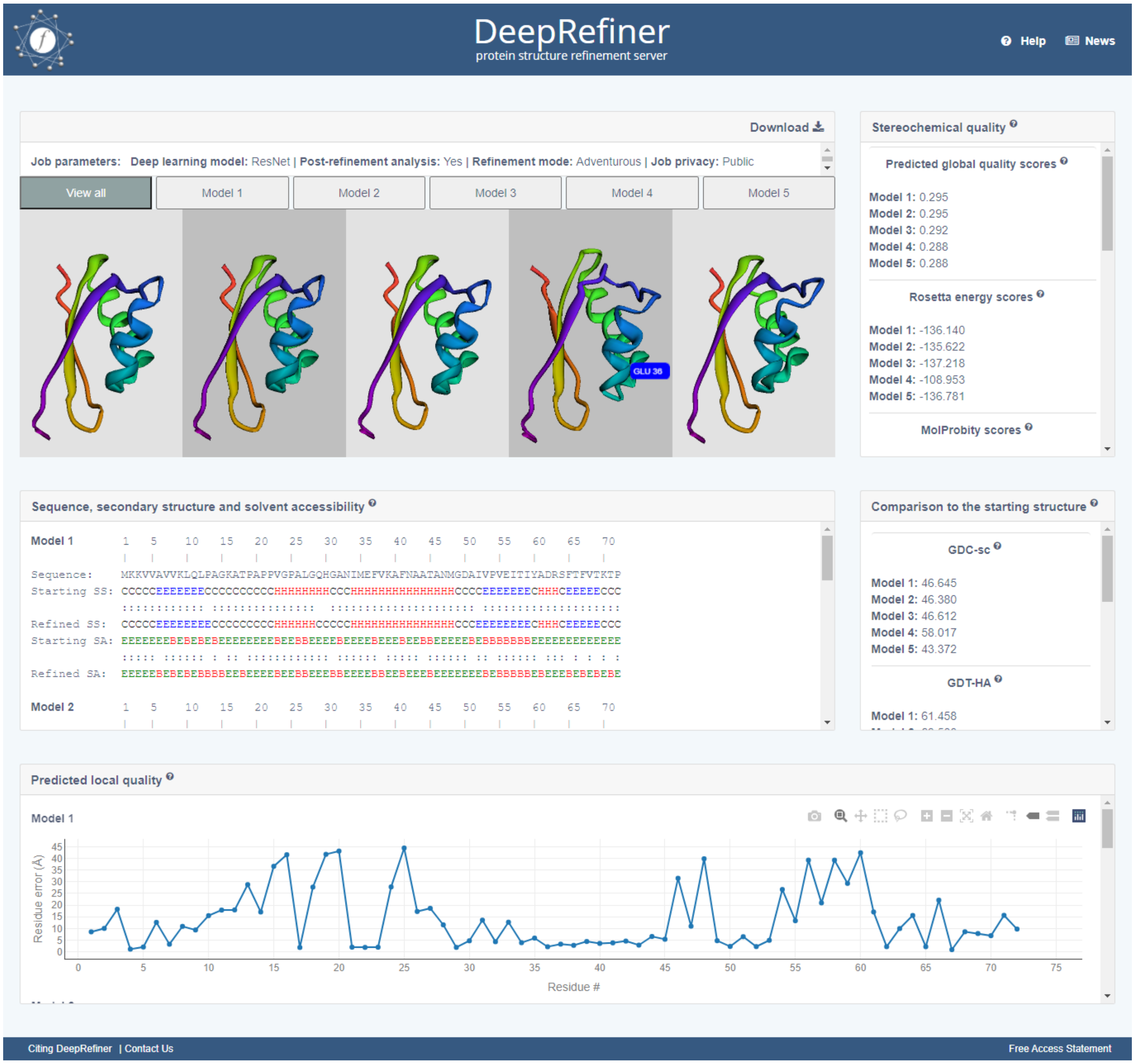

Figure S1. Interactive quantitative and visual analysis of the refinement results by DeepRefiner.

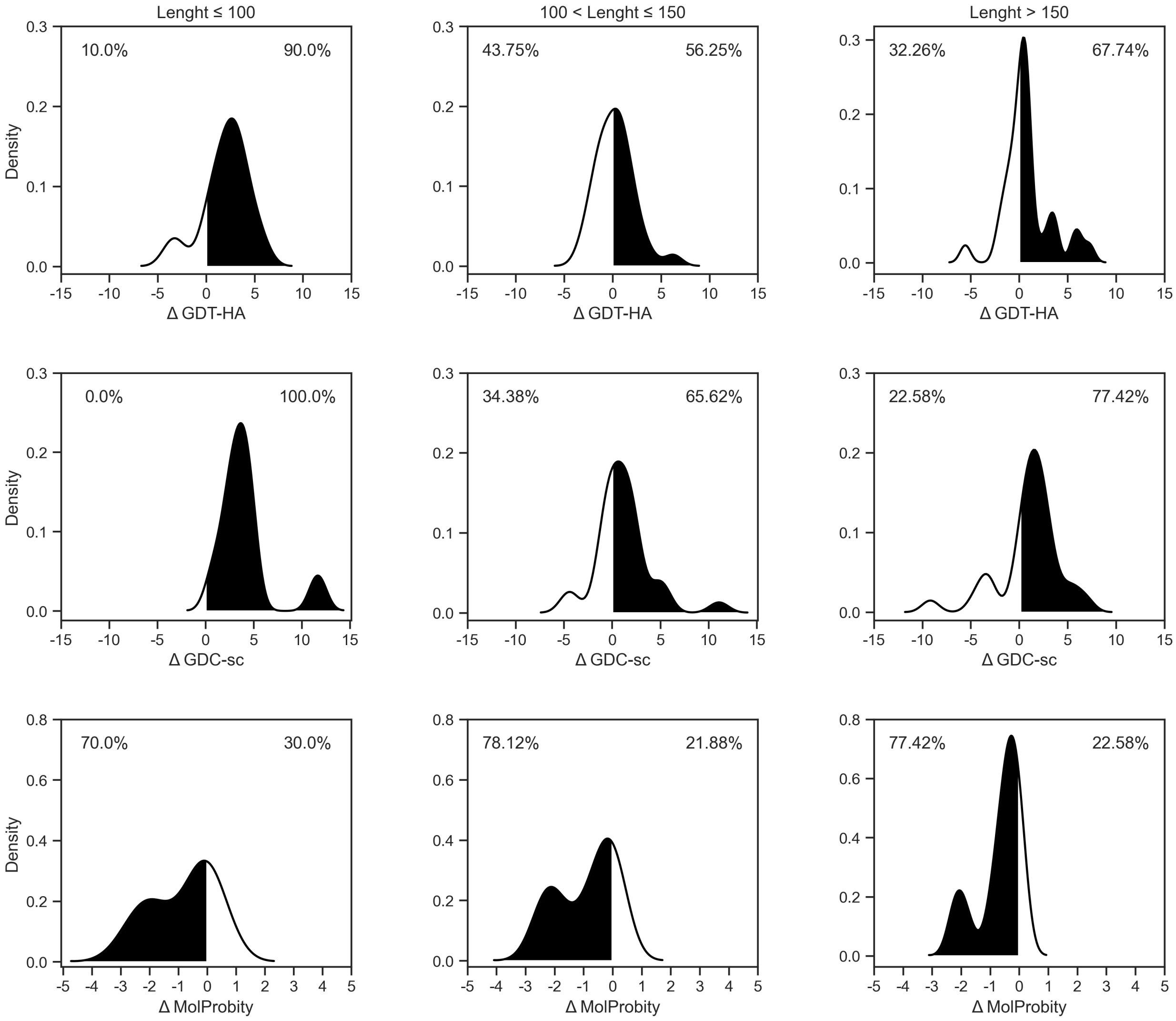

Figure S2. Degree of refinement for "Bhattacharya-Server" considering all CASP13 and CASP14 refinement targets grouped by various length bins. Distributions of ΔGDT-HA, ΔGDC-sc, and ΔMolProbilty scores for various length bins considering the best submission presented. Regions shaded in black illustrate improvement over the starting structure. Numbers above the distribution plots indicate the percentages of refinement successes and failures.

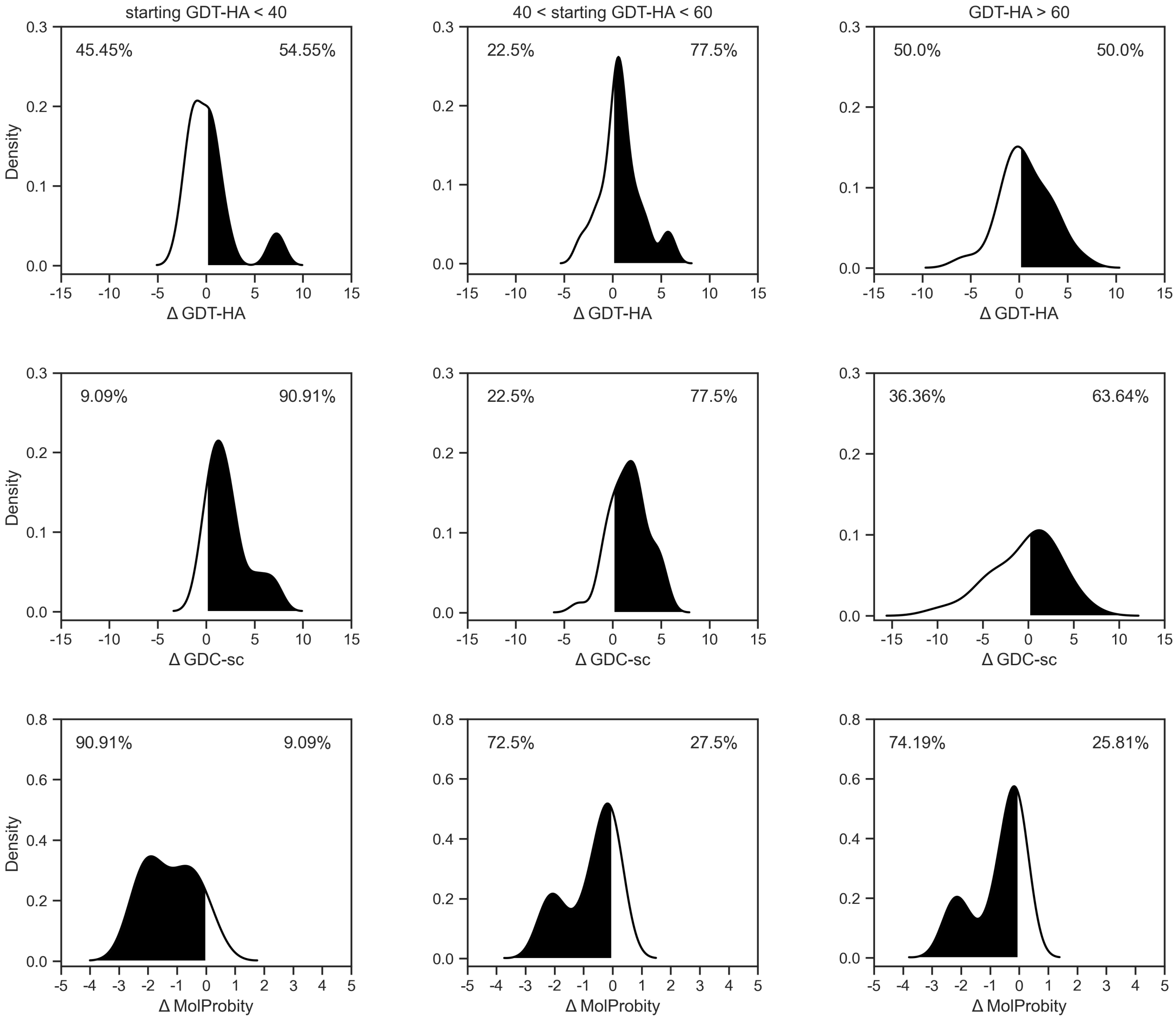

Figure S3. Degree of refinement for "Bhattacharya-Server" considering all CASP13 and CASP14 refinement targets grouped by various starting GDT-HA bins. Distributions of ΔGDT-HA, ΔGDC-sc, and ΔMolProbilty scores for various starting GDT-HA bins considering the best submission presented. Regions shaded in black illustrate improvement over the starting structure. Numbers above the distribution plots indicate the percentages of refinement successes and failures.

References

1. Remmert,M., Biegert,A., Hauser,A. and Söding,J. (2012) HHblits: lightning-fast iterative protein sequence searching by HMM-HMM alignment. *Nat Methods*, **9**, 173–175.
2. Mirdita,M., von den Driesch,L., Galiez,C., Martin,M.J., Söding,J. and Steinegger,M. (2017) Uniclust databases of clustered and deeply annotated protein sequences and alignments. *Nucleic Acids Res*, **45**, D170–D176.
3. Altschul,S.F., Madden,T.L., Schäffer,A.A., Zhang,J., Zhang,Z., Miller,W. and Lipman,D.J. (1997) Gapped BLAST and PSI-BLAST: a new generation of protein database search programs. *Nucleic Acids Res*, **25**, 3389–3402.
4. Heffernan,R., Paliwal,K., Lyons,J., Dehzangi,A., Sharma,A., Wang,J., Sattar,A., Yang,Y. and Zhou,Y. (2015) Improving prediction of secondary structure, local backbone angles, and solvent accessible surface area of proteins by iterative deep learning. *Sci Rep*, **5**, 11476.
5. Kabsch,W. and Sander,C. (1983) Dictionary of protein secondary structure: Pattern recognition of hydrogen-bonded and geometrical features. *Biopolymers*, **22**, 2577–2637.
6. Heinig,M. and Frishman,D. (2004) STRIDE: a web server for secondary structure assignment from known atomic coordinates of proteins. *Nucleic Acids Res*, **32**, W500–W502.
7. Greener,J.G., Kandathil,S.M. and Jones,D.T. (2019) Deep learning extends de novo protein modelling coverage of genomes using iteratively predicted structural constraints. *Nat Commun*, **10**, 1–13.
8. Li,Y., Hu,J., Zhang,C., Yu,D.-J. and Zhang,Y. (2019) ResPRE: high-accuracy protein contact prediction by coupling precision matrix with deep residual neural networks. *Bioinformatics*, **35**, 4647–4655.
9. Leaver-Fay,A., Tyka,M., Lewis,S.M., Lange,O.F., Thompson,J., Jacak,R., Kaufman,K., Renfrew,P.D., Smith,C.A., Sheffler,W., *et al.* (2011) Rosetta3: An Object-Oriented Software Suite for the Simulation and Design of Macromolecules. *Methods Enzymol*, **487**, 545–574.
10. Rohl,C.A., Strauss,C.E.M., Misura,K.M.S. and Baker,D. (2004) Protein structure prediction using Rosetta. *Meth. Enzymol.*, **383**, 66–93.
11. Bhattacharya,D. (2019) refineD: improved protein structure refinement using machine learning based restrained relaxation. *Bioinformatics*, **35**, 3320–3328.
12. Shuvo,M.H., Bhattacharya,S. and Bhattacharya,D. (2020) QDeep: distance-based protein model quality estimation by residue-level ensemble error classifications using stacked deep residual neural networks Bioinformatics.
13. He,K., Zhang,X., Ren,S. and Sun,J. (2016) Deep Residual Learning for Image Recognition. In *2016 IEEE Conference on Computer Vision and Pattern Recognition (CVPR)*.pp. 770–778.
14. Ng,A.Y. (2004) Feature selection, L_1 vs. L_2 regularization, and rotational invariance. In *Twenty-first international conference on Machine learning - ICML '04*. ACM Press, Banff, Alberta, Canada, p. 78.
15. Zemla,A. (2003) LGA: a method for finding 3D similarities in protein structures. *Nucleic Acids Res*, **31**, 3370–3374.
16. Chollet,F. (2015) Keras: Deep learning library for theano and tensorflow.
17. Alford,R.F., Leaver-Fay,A., Jeliazkov,J.R., O'Meara,M.J., DiMaio,F.P., Park,H., Shapovalov,M.V., Renfrew,P.D., Mulligan,V.K., Kappel,K., *et al.* (2017) The Rosetta All-Atom Energy Function for Macromolecular Modeling and Design. *J. Chem. Theory Comput.*, **13**, 3031–3048.
18. Khatib,F., Cooper,S., Tyka,M.D., Xu,K., Makedon,I., Popović,Z., Baker,D. and Players,F. (2011) Algorithm discovery by protein folding game players. *PNAS*, **108**, 18949–18953.
19. Tyka,M.D., Keedy,D.A., André,I., Dimaio,F., Song,Y., Richardson,D.C., Richardson,J.S. and Baker,D. (2011) Alternate states of proteins revealed by detailed energy landscape mapping. *J. Mol. Biol.*, **405**, 607–618.
20. Kopp,J., Bordoli,L., Battey,J.N.D., Kiefer,F. and Schwede,T. (2007) Assessment of CASP7 predictions for template-based modeling targets. *Proteins*, **69 Suppl 8**, 38–56.
21. Kabsch,W. (1976) A solution for the best rotation to relate two sets of vectors. *Acta Crystallographica Section A*, **32**, 922–923.
22. MacCallum,J.L., Hua,L., Schnieders,M.J., Pande,V.S., Jacobson,M.P. and Dill,K.A. (2009) Assessment of the protein-structure refinement category in CASP8. *Proteins: Structure, Function, and Bioinformatics*, **77**, 66–80.
23. Kryshtafovych,A., Monastyrskyy,B. and Fidelis,K. (2014) CASP PREDICTION CENTER INFRASTRUCTURE AND EVALUATION MEASURES IN CASP10 AND CASP ROLL. *Proteins*, **82**, 7–13.
24. Chen,V.B., Arendall,W.B., Headd,J.J., Keedy,D.A., Immormino,R.M., Kapral,G.J., Murray,L.W., Richardson,J.S. and Richardson,D.C. (2010) MolProbity: all-atom structure validation for macromolecular crystallography. *Acta Crystallogr D Biol Crystallogr*, **66**, 12–21.
25. Hovan,L., Oleinikovas,V., Yalinca,H., Kryshtafovych,A., Saladino,G. and Gervasio,F.L. (2018) Assessment of the model refinement category in CASP12. *Proteins*, **86 Suppl 1**, 152–167.
26. Nugent,T., Cozzetto,D. and Jones,D.T. (2014) Evaluation of predictions in the CASP10 model refinement category. *Proteins*, **82 Suppl 2**, 98–111.
